# Contribution of CNS cells in NeuroAIDS

**DOI:** 10.4103/0975-7406.72129

**Published:** 2010

**Authors:** Ashish Swarup Verma, Udai Pratap Singh, Premendra Dhar Dwivedi, Anchal Singh

**Affiliations:** Amity Institute of Biotechnology, Amity University Uttar Pradesh, Sector -125, Noida (UP) - 201 303, India; 1 Food and Dye Toxicology, Indian Institute of Toxicology Research, PO Box - 80, MG Marg, Lucknow - 226 001, UP, India

**Keywords:** Central nervous system, HIV, NeuroAIDS

## Abstract

NeuroAIDS is becoming a major health problem among AIDS patients and long-term HIV survivors. As per 2009 estimates of UNAIDS report, more than 34 million people have been infected with HIV out of which ≥ 50% show signs and symptoms of neuropsychiatric disorders. These disorders affect central nervous system (CNS) and peripheral nervous systems (PNS). CNS is one of the most protected organ systems in body which is protected by blood-brain barrier (BBB). Not only this, most of the cells of CNS are negative for receptors and co-receptors for HIV infections. Neurons have been found to be completely nonpermissive for HIV infection. These facts suggest that neurotoxicity could be an indirect mechanism responsible for neuropsychiatric complications. In this review, we will discuss the importance of different cell types of CNS and their contribution toward neurotoxicity.

Neuropsychiatric complications among AIDS patients are called NeuroAIDS. A combination of neurological and psychiatric disorders is considered as neuropsychiatric disorder which includes mood disorders, schizophrenia, addiction, dementia, epilepsy, etc. Neuropsychiatric disorders contribute ~15% of total burden of world’s disease.[1,2] An abrupt increase in incidences of neuropsychiatric disorders has been observed among HIV seropositives as well as AIDS patients in last 10 years.

More than 25 years ago, HIV was reported as a unique infection with certainty of death. Initially, the main objective of treatment was to control HIV infections and death associated with it. Therefore, an expected attention was not paid to neurological and psychiatric complications noticed among HIV patients. Incidences of opportunistic infections along with baffling signs and symptoms of neurological disorders could be traced as even in the first known report about HIV/AIDS patients.[3,4] Some of the common opportunistic infection in AIDS/HIV seropositive has been listed in Table 1. Undoubtedly, medications for HIV treatment have seen enormous improvements, which have enhanced the survival among HIV seropositives. Some HIV seropositives even survive for more than 20 years after initial exposure to HIV. The current evidences clearly suggest that variety of neurological and psychological disorders among early patients of HIV/AIDS were indicative of their late stages of HIV infections.

**Table 1:** Commonly occurring opportunistic infections in HIV/AIDS patients

Disease	Infectious organism
Pneumonia	*Pneumocystis carinii*
Pneumonia	*Pneumocystis jiroveci*
Kaposi’s sarcoma	Kaposi’s sarcoma virus (KSV)
Tuberculosis	*Mycobacterium avium*
Tuberculosis	*Mycobacterium tuberculosis*
Cryptococcal meningitis	*Cryptococcus neoformans*
Toxoplasmic encephalitis	*Toxoplasma gondii*
Progressive multifocal leukoencephalopathy	J C Virus (JCV)
Cytomegalovirus encephalitis	Cytomegalovirus (CMV)

Importance of HIV infection can be gauged with the fact that ~6800 patients get infected with HIV, while ~5700 patients die with AIDS everyday.[5] As per current estimate, >34 million people have been infected with HIV out of which ≥50% of HIV seropositives show symptoms of neurological complications. These neurological complications are associated either with central nervous system (CNS) or peripheral nervous system (PNS) or both.[6] Generally these symptoms become more clinically evident with the progression of AIDS. AIDS can be clinically defined when (i) condition in which CD4 ^+^ T-lymphocyte counts are <200 cells/*μ*l of blood, (ii) presence of AIDS defining illnesses like HAD (HIV-associated dementia), HIV wasting syndrome, *etc*.[6,7]

Unfortunately, neuroAIDS afflicts patients at their prime age i.e., ~30–40 years, resulting in a major loss of human productivity. Neuropsychiatric disorders on the one hand lead to increase in health care cost for the patient, while on the other hand these patients become ineffective to perform any productive work either for self or for society.[8,9]

HIV variations at molecular level are described as clades. Clades have geographical preferences, *e.g*., clade B is prevalent in industrialized world while other clade types like A, C, E, *etc*. are prevalent mostly in developing or underdeveloped regions. Mixing as well as evolution of these clades have also been observed.[10] Apart from clade variation, drug resistance is also common in occurrence.[11] Importance of evolution of drug resistant HIV cannot be denied for its impact on AIDS as well as neuroAIDS. Majority of data about neuroAIDS has come from clade B of HIV. Knowledge about the role of other clades is very limited because (i) patients infected with clades other than clade B rarely have life-long access to antiretroviral treatments (ART) and (ii) these clades are prevalent in societies lacking resources.

Improved antiretrovirals have reduced morbidity and mortality among HIV seropositives.[12,13] An increase in life span possibly leads toward neuroAIDS among long-term HIV survivors. In current circumstances, a significant increase in neuroAIDS cases cannot be ignored, as it may have serious consequences in near future.

Neurotoxicity is the major reason for the onset of neurocognitive disorders among HIV seropositives. Neuropsychiatric disorders among HIV patients such as HIV-associated dementia (HAD), HIV-associated encephalopathy (HIVE), *etc*. are common with wide-ranging symptoms [Table 2]. Neurotoxicity is considered as the major contributing factor for neuroAIDS. Still it is debatable, “What are the triggers for neurotoxicity?” Some of the possibilities are under active considerations such as (i) Is HIV itself responsible for neuroAIDS? (ii) Is neuroAIDS a secondary complication evolved due to long-term ART? (iii) Are low levels of persistent and chronic HIV infections contributing toward the development of neuroAIDS? Or (iv) Is it a combined effect of all these possibilities? The present understanding of neuroAIDS and its causes are still not very clear!

**Table 2:** Common neuropsychiatric disorders in HIV/AIDS patients

Neuropsychiatric disorders
Addiction
Anxiety
Depression
Epilepsy
Mania
Mood disorders
Neurocognitive impairment
Neuropathic pain
Physical disability
Seizure

In this article we will discuss susceptibility of various cell types of CNS for HIV infection and their implications in neuropsychiatric disorder among HIV seropositives.

## Receptors and Co-Receptors for HIV Infection

CD4 is a primary receptor for HIV infection and is commonly found on peripheral blood mononuclear cells especially on T-cells, monocytes, and macrophages.[11,14] Other co-receptors for HIV infection have also been identified which are known as chemokine receptors. Chemokine receptors are of two types: CXCR4 and CCR5. Expression of CD4 receptors on brain cells is from negligible to none. Most common cells of CNS, *viz*., oligodendrocytes and neurons, are negative for CD4 antigen. Microglia and perivascular macrophages are the only two cell types that express CD4 antigens [Table 3].

**Table 3:** HIV infectivity and cells of central nervous system

Cell type	CD4 receptor	Chemokine receptor	HIV susceptibility	Productive infections
Astrocytes	−	+	Yes	−
Brain microvascular endothelial cells	−	+	*in vitro*	−
Microglia	+	+	Yes	+
Neurons	−	+	No	−
Oligodendrocytes	−	+	*in vitro*	−
Perivascular macrophages	+	+	Yes	+

Importance of co-receptors cannot be denied for their role in HIV infection of CNS. Chemokines and their receptors have been reported to play a role in pathogenesis of different neurological disorders like HAD, Alzheimer’s disease, prion infection, *etc*. Over-expression or pathological expression of chemokines may cause pathogenesis in specific brain areas. Therefore, chemokine receptors may also serve as an entry point for HIV infection into CNS.[15] CXCR4 receptors are expressed on neurons of brain of HAD patients.[16] Hippocampal neurons in HAD have been reported to be positive for CCR2, CCR3, and CCR4.[17] Microglia and neuron were also found positive for CCR2, CCR3, and CXCR4 in HAD. These chemokines are highly expressed in sub-cortical and limbic region, out of which limbic region is considered mainly responsible for cognitive functions. Probably, this could be one of the reasons for cognitive disorder and memory dysfunctions among HAD patients. An elevated level of CCR1, CCR3, CCR5, and CXCR4 has been reported in microglial nodules.[18,19] An over-expression of CX3C chemokine, fractalkine/CXC3CLI were reported in pediatric patients, while overexpression of fractalkine/CX3CLI have been reported in adult patients.[20,21] Chemokine levels have been positively correlated with severity of dementia and viral load, which is an indicator of their significance in HIV- induced neuropathogenesis.

A decline in cognitive, motor function, as well as behavioral disorders among HAD patient can be explained by a significant increase in neuronal death, which could be a consequence of HIV infection to brain.[22,23] HIV infections to neurons are rare to none; therefore neuronal loss is possibly due to neurotoxic factors produced by HIV-infected cells of CNS, *viz*., astrocytes, microglia, or other neurotoxic proteins of HIV origin.

## Neuropathogenesis of HIV

CNS is considered as one of the most protected organ/system in body because of the presence of protective layer called as blood-brain barrier (BBB). BBB is a continuous layer of tightly linked microvascular endothelial cells surrounding brain, which separates CNS from rest of the body. The cerebrospinal fluid (CSF) is separated from peripheral circulation by the blood-CSF barrier known as choroid plexus.[24] Pathological conditions and injuries to CNS leave opportunities for pathogens, immune cells, and various biomolecules to enter into CNS. CNS majorly comprises of five different types of cells, *viz*., astrocytes, oligodendrocytes, neurons, microglia, and perivascular macrophages.[25,26]

## Neurons

Neurons are main effector cells for cognitive and motor functions. Therefore, importance of neurons in HIV related neuropathogenesis cannot be ignored. A significant neuronal death has been reported in HIV infected brains[27–29] even though neurons are non-permissive for HIV infections. Most of the studies have failed to demonstrate HIV infections *in vivo*; however few studies have reported presence of HIV viral DNA and proteins in neurons.[30,31] Neurons do not express receptors for HIV infection, which is the strongest argument for neuronal non-permissiveness for HIV. A strain restricted infection to primary neurons[32] and to neuronal cell lines have been reported *in vitro*; however, same HIV strains failed to infect neurons *in vivo*.[33–35] The major reason for failure to demonstrate HIV infection in neuronal cells is due to inherent qualities of neurons itself. Neurons in response to adverse stimulus tend to die either by apoptosis or necrosis. Therefore, a productive HIV infection in neurons is almost impossible even *in vitro*. Inability of neurons to regenerate may also be a contributing factor for the failure to detect any HIV infections.[25]

## Oligodendrocytes

Oligodendrocytes produce myelin sheath, which supports fast axonal conduction. An alteration in axonal conduction is possible during HIV infections. Oligodendrocytes are CD4 negative cells but they express chemokine receptors, which raises the question, “How can oligodendrocytes get infected with HIV?” Absence of receptors also supports non-infectivity of oligodendrocytes. Some studies have shown the presence of HIV nucleic acid in oligodendrocytes by in situ PCR, while another study has reported absence of any markers of HIV infection.[30,31,36] A limited HIV infectivity has been reported *in vitro* with specific HIV strain.[37] Binding of HIV gp120 to galactosylceramide or other proteoglycans of oligodendrocytes may reduce myelin synthesis and can cause increase in intracellular Ca^2+^ levels and apoptosis, which further contribute towards HIV neuropathogenesis.[38]

## Microglia and Perivascular Macrophages

Astrocytes, microglia, and perivascular macrophages are the major cell types found in perivascular region of brain. Microglia and perivascular macrophages act as resident immunocompetent cells and respond to any insult to brain (including infections). These cells also respond upon entry of infected cells including HIV-infected monocytes and T-cells. HIV-infected monocytes and T-cells enter into brain due to breach in BBB. Peripheral migratory monocytes (infected) after entry into brain differentiate into monocyte-derived macrophages of CNS (or also known as perivascular macrophages) and these cells are considered as a possible source for productive HIV infection in brain.[39,40]

Microglial cells have been reported to be immuno-positive for HIV. HIV replication in primary microglial cells from adults, infants, as well as fetal brain have been demonstrated *in vitro*.[41–45] Microglial cells functions are similar to macrophages and they express major receptors and co-receptors for HIV infections like CD4, CCR5, along with other chemokine receptors, *viz*., CCR3, CCR2b, CCR8, CXCR6, and CX3CR1.[18,46–48] Presence of these receptors and co-receptors makes microglial cells vulnerable for HIV infection. Microglial cells possibly act as a source for productive HIV infections under the influence of different pro-inflammatory and inflammatory cytokines. *In vitro* studies have demonstrated that mixed microglial culture from human brain can retain replication competent HIV up to few months, although HIV replication is of low level.[49]

Perivascular macrophages are CD4^+^45^+^, flat and elongated cells mostly located adjacent to brain microvasculature endothelial cells. The turnover rates of perivascular macrophages are higher compared to microglia, due to its closer proximity to peripheral circulation. Perivascular macrophages get regularly replenished from peripheral monocytes. This replenishment could be considered as side effect of “opening the door” phenomenon. In case of HIV-1 and HIV-2 infections, perivascular macrophages are some of the most infected cells, which has also been confirmed by in situ immunohistochemistry, while in simian immunodeficiency virus (SIV), these cells have also shown active viral replication.[50–52]

## Astrocytes

Astrocytes maintain homeostasis of CNS and express receptors for various neuroreactive compounds including neurotransmitters. Astrocytes act as sentinels by regulating levels of neurotransmitters like glutamate. A proliferative response among astrocytes has been reported in HIV-infected brains.[53,54] Astrocytes have failed to show any robust viral replication because only few astrocytes were found to be positive for HIV antigen. The expression of CD4 antigen in astrocytes is subminimal or negligible. Therefore, mechanism for HIV entry into astrocytes is questionable. In HIV patients, astrocytosis has been reported in response to viral proteins or other macrophage products. It has been implicated that astrocytosis may have a role in HIV-induced neuropathogenesis.[50,51,55]

## Trojan Horse Hypothesis for Neuroinvasion

Penetration of HIV into CNS is known as “Neuroinvasion.” Neuroinvasion to CNS is still quite controversial because (i) HIV receptors and co-receptors are generally not expressed in brain cells except microglia and perivascular macrophages, (ii) CNS is protected by a unique protective layer known as BBB, which acts as sentry for brain, and (iii) any adverse response to neuron leads to neuronal cell death either by necrosis or by apoptosis.

**Figure 1:**
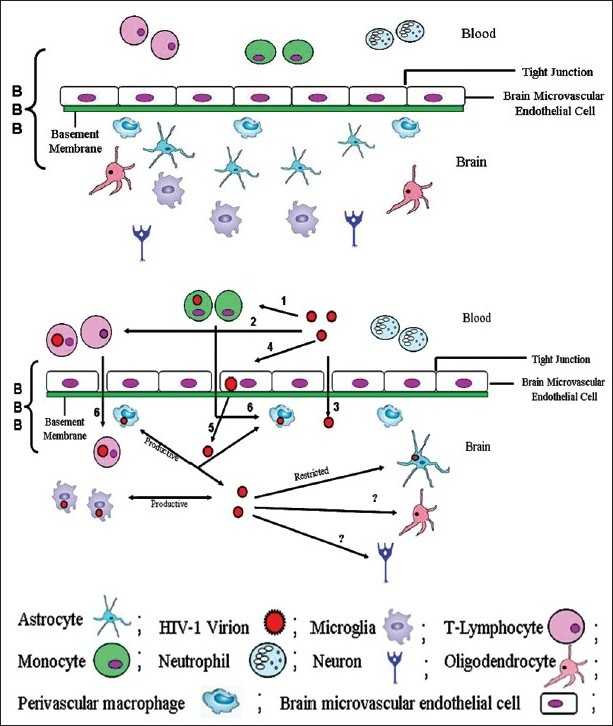
Neuroinvasion of HIV-1: the figure depicts cellular components of blood–brain barrier (BBB). 1a) A normal BBB is represented here. It contains different cell types, *viz*., astrocytes, microglia, microvascular endothelial cells, oligodendrocytes, perivascular macrophages, *etc*. 1b) This figure is a graphical representation of various means for HIV entry into CNS including “Trojan horse hypothesis”. (1 and 2) HIV infection to monocytes and T-lymphocytes in peripheral blood circulation. Upon HIV infection there can be breaches in BBB where (3) HIV may directly enter into brain via opening in tight junction, (4 & 5) HIV entry into the brain by transcytosis phenomenon, (6) HIV-infected monocytes and T-lymphocytes acts as carrier for HIV infections to the brain, and (?) mechanism of HIV infection to neurons and oligodendrocytes is still questionable

BBB has selective permeability and it regulates trafficking of cells and other substances, which crosses BBB [Figure 1]. Any foreign material (including HIV) has to cross BBB to enter into brain; however, the mechanism for its entry into CNS is still not clear. Various animal models as well as *in vitro* experiments have been tried to understand mechanism of HIV entry into CNS via BBB.[25,26]

On the basis of different experimental evidences, various models have been proposed for neuroinvasion of HIV. They are (i) direct entrance of virus into brains, (ii) transcytosis, and (iii) Trojan horse hypothesis. Out of these three hypotheses, “Trojan horse hypothesis” is the most acceptable model to explain HIV infections to CNS.[56,57]

As per Trojan horse hypothesis, HIV enters into CNS as a passenger with infected cells finding their entry into brain. There are various CD4^+^ cells like T-cells and monocytes which are already infected with HIV, and freely circulate into blood circulation. These infected cells cross BBB and reach CNS where they may be responsible to propagate HIV infection to other CNS cells.[56] Evidences from *in situ* hybridization and immunohistochemical staining support Trojan horse hypothesis model with the accumulation of HIV in perivascular macrophages.[50–52] HIV-induced abnormalities in BBB has been observed but mechanism of microvascular endothelial cell infection is still not clear as these cells lack receptors and co-receptors for HIV infections. These cells express CCR5 and CXCR4, while expression of CD4 on these cells is contradictory.[58–60] Other ways of HIV entry into brain are “direct entry” to CNS or by “transcytosis” through microvascular endothelial cells.[25,61–63]

## Inflammatory Cascade

Neurons do not get infected with HIV, even though; neurodegeneration and neuronal loss have been commonly observed in HIV seropositives. Neurotoxicity must be a causative factor for this neuronal loss. Neurotoxicity may be induced by excessive production of inflammatory biomolecules or mediators of inflammation produced by different cell types of CNS.

Monocytes, lymphocytes, and activated macrophages after entering into CNS release various pro-inflammatory, inflammatory cytokines, reactive oxygen, and other biomolecules with high neurotoxic potential. These mediators individually, additively, or synergistically disrupt normal functioning of cells of CNS by inducing neurotoxicity. This may cause alterations in neurotransmitter action and causes leukoencephalopathy resulting in neuronal apoptosis.[64,65] TNF-α, platelet activating factor (PAF), nitric oxide (NO), and quinolinic acid (QUIN) also behave like neurotoxicant and cause neurotoxicity. NO is produced by microvascular endothelial cells, macrophages, and neurons which may result in N-methyl-D-aspartate (NMDA) type glutamate-associated neurotoxicity. Elevated levels of NO synthase has been reported in the brain of HAD patients, while a 40-fold increase in expression of NO synthase in neurons of drug addict HIV patients.[66,67] TNF-α is produced by macrophages and microglia and it mainly affects oligodendrocytes.[68] An elevated level of TNF-α mRNA has been reported in HIV patients with neurological complications.[69] TNF-α causes damage to BBB and facilitates entry of peripheral blood cells.[70] Pro-inflammatory cytokines like TNF-α, IL-1, and IFN-α are found to be present in elevated level in AIDS patients.[71,72]

## HIV Compartmentalization

T-cells and monocytes are primary target for HIV infection because they are positive for CD4 and co-receptors. These cells are always a source of productive HIV infection. In turn, these infections may become a source for CNS infection. At what stage HIV infects CNS is still a question, as there are no conclusive evidences for this. Although, the various possibilities for CNS infection are as follows: (i) HIV infects CNS soon after the infection of peripheral blood cells, (ii) HIV infection in CNS remains latent for a longer time after initial infection, (iii) HIV infections get cleared from CNS after initial infection and re-enter in CNS at a later stage during the course of infection, (iv) CNS gets infected with HIV at different time points, post-HIV infections. HIV infections of CNS at different time points during course of HIV infection seem to be most rationale scenario among all these possibilities.[73]

HIV infection can be compartmentalized into two compartments: (A) peripheral tissues which include blood, lymphnodes, and spleen, where mutation of HIV or phylogenetic evolution of HIV is rapid and high, while (B) CNS, which is a separate compartment for HIV replication where HIV replication is comparatively slow. Phylogenetic studies have shown that viruses present in CNS are more closely related to each other suggestive of slow mutation rate in CNS.[74–78] The reasons for this slow mutation rate in CNS is due to (i) low penetrance of antiretroviral drugs to CNS leaving minimal chances for development of drug resistance, (ii) a reduced production of neutralization antibody in CNS, and (iii) reduced response of cytolytic T-cells in CNS. Another contributing factor for this restricted HIV replication and close resemblance in HIV progeny from CNS is due to unavailability of CNS cells, which can support HIV replication. Oligodendrocytes and neurons do not participate directly in HIV replication with exception of astrocytes and perivascular macrophages. But, the participation of all these cell types in neurotoxicity is indirect because the ability of these cells to remove toxic products from CNS is significantly diminished. Majority of CNS cells are incapable of productive HIV infection with the exception of microglia and perivascular macrophages.

## Future Perspective

NeuroAIDS is expected to be a major upcoming health issue among long-term HIV seropositive survivors and AIDS patients. Microglial cells and perivascular macrophages are known to be infected with HIV, while neurons are completely nonpermissive to HIV infections. Neuronal loss is commonly observed among AIDS patients, even though neurons are nonpermissive to HIV infections. This neuronal loss could be triggered due to direct/indirect neurotoxicity induced due to infection of other cells of CNS. Majority of data obtained about neuropathogeneis of HIV has been obtained either *in vitro* studies or from cadaver samples, as we do not have any suitable animal model for HIV. It is difficult to judge the role of HIV infection and accessory biomolecules on neurocognitive impairment because of non-availability of any suitable animal model either for HIV or neuroAIDS. Therefore, it is very important to develop an animal model for neuroAIDS to study the impact of HIV infections to CNS and its consequences. Various anti-inflammatory agents, drug-efflux pump inhibitors, and even nanoparticles have shown their significance with therapeutic potential. But their efficacy for neuroAIDS can be established with testing in improved *in vitro* models for BBB as well as with the search of a suitable animal model for neuroAIDS. This will also help us to elucidate the mechanism and interaction of various cell types of CNS during active infection and replication in CNS.

## References

[1] Prince M, Patel V, Saxena S, Maj M, Maselko J, Phillips MR (2007). No health without mental health. Lancet.

[2] McCombe JA, Noorbakhsh F, Buchholz C, Trew M, Power C (2009). NeuroAIDS : A watershed for mental health and nervous system disorders. J Psychiatry Neurosci.

[3] Anthony IC, Bell JE (2008). Neuropathology of HIV/AIDS. Int Rev Psychiatry.

[4] Gottlieb MS, Schroff R, Schanker HM, Weisman JD, Fan PT, Wolf RA (1981). *Pneumocystis carinii* pneumonia and mucosal candidiasis in previously healthy homosexual men: Evidence of a new acquired cellular immunodeficiency. N Engl J Med.

[5] (2009). AIDS epidemic update. UNAIDS Report.

[6] Power C, Boisse L, Rornke S, John GM (2009). NeuroAIDS: An evolving epidemic. Can J Neurosci.

[7] (1992). Centers for Disease Control and Prevention. 1993 revised classification system for HIV infection and expanded surveillance definition for AIDS among adolescents and adults. MMWR.

[8] Patrick MK, Johnston JB, Power C (2002). Lentiviral neuropathogenesis: Comparative neuroinvasion, neurotropism, neurovirulence, and host neurosusceptibility. J Virol.

[9] Pandya R, Krentz HB, Gill MJ, Power C (2005). HIV-related neurological syndromes reduce health-related quality of life. Can J Neurol Sci.

[10] Spira S, Wainberg MA, Loemba H, Turner D, Brenner BG (2003). Impact of clade diversity on HIV-1 virulence, anti retroviral drugs, sensitivity and drug resistance. J Antimicrob Chemother.

[11] Verma AS, Bhatt SM, Singh A, Diwedi PD, Varma A, Chauhan AK (2009). HIV: An introduction. Text book on molecular biotechnology.

[12] (2004). WHO/UNAIDS. 3 by 5 progress report, December 2004.

[13] Weungi H, Krentz HB, Gill MJ, Power C (2006). Neuropsychiatric disorders in HIV infection: Impact of diagnosis on economic cost of care. AIDS.

[14] Abbas AK, Lichtman AH, Pillai S (2008). Cellular and molecular immunology.

[15] Li W, Galey D, Mattson MP, Nath A (2005). Molecular and cellular mechanisms of neuronal cell death in HIV dementia. Neurotox Res.

[16] Dou H, Kingsley JD, Mosley RL, Gelbard HA, Gendelman HE (2004). Neuroprotective strategies for HIV-1 associated dementia. Neurotox Res.

[17] Vander Meer P, Ulrich AM, Gonzalez-Scarano F, Lavi E (2000). Immunohistochemical analysis of CCR2, CCR3, CCR5, and CXCR4 in the human brain: Potential mechanisms for HIV dementia. Exp Mol Pathol.

[18] Vallat AV, De Girolami U, He J, Mhashilkar A, Marasco W, Shi B (1998). Localization of HIV-1 co-receptors CCR5 and CXCR4 in the brain of children with AIDS. Am J Pathol.

[19] Sanders VJ, Pittman CA, White MG, Wang G, Wiley CA, Achim CL (1998). Chemokines and receptors in HIV encephalitis. AIDS.

[20] Tong N, Perry SW, Zhang Q, James HJ, Guo H, Brooks A (2000). Neuronal fractalkine expression in HIV-1 encephalitis: Roles for macrophage recruitment and neuroprotection in the central nervous system. J Immunol.

[21] Pereira CF, Middel J, Jansen G, Verhoef J, Nottet HS (2001). Enhanced expression of fractalkine in HIV-1 associated dementia. J Neuroimmunol.

[22] Masliah E, Achim CL, Ge N, DeTeresa R, Terry RD, Wiley CA (1992). Spectrum of human immunodeficiency virus-associated neocortical damage. Ann Neurol.

[23] Petito CK, Roberts B (1995). Effect of postmortem interval on *in situ* end-labeling of DNA oligonucleosomes. J Neuropathol Exp Neurol.

[24] Rapoport SI (1976). Blood-Brain Barrier in Physiology and Medicine.

[25] Gonzalez-Scarano F, Martin-Garcia J (2005). The neuropathogenesis of AIDS. Nat Rev Immun.

[26] Ghafouri M, Amini S, Khalili K, Sawaya BE (2006). HIV-1 associated dementia: Symptoms and causes. Retrovirology.

[27] Adie-Biassette H, Levy Y, Colombel M, Poron F, Natcher S, Keohane C (1995). Neuronal apoptosis in HIV infection in adults. Neuropathol Appl Neurobiol.

[28] Gelbard HA, James HJ, Sharer LR, Perry SW, Saito Y, Kazee AM (1995). Apoptotic neurons in brains from paediatric patients with HIV-1 encephalitis and progressive encephalopathy. Neuropathol Appl Neurobiol.

[29] Petito CK, Roberts B (1995). Evidence of apoptotic cell death in HIV encephalitis. Am J Pathol.

[30] Bagasra O, Lavi E, Bobroski L, Khalili K, Pestaner JP, Tawadros R (1996). Cellular reservoirs of HIV-1 in the central nervous system of infected individuals: Identification by the combination of *in situ* polymerase chain reaction and immunohistochemistry. AIDS.

[31] Nuovo GJ, Becker J, Burk MW, Margiotta M, Fuhrer J, Steigbigel RT (1994). *In situ* detection of PCR-amplified HIV-1 nucleic acids in lymph nodes and peripheral blood in patients with asymptomatic HIV-1 infection and advanced-stage AIDS. J Acquir Immune Defic Syndr.

[32] Ensoli F, Cafaro A, Fiorelli V, Vannelli B, Ensoli B, Thiele CJ (1995). HIV-1 infection of primary human neuroblasts. Virology.

[33] Obregon E, Punzon C, Fernandez-Cruz E, Fresno M, Munoz-Fernandez MA (1999). HIV-1 infection induces differentiation of immature neural cells through autocrine tumor necrosis factor and nitric oxide production. Virology.

[34] Mizrachi Y, Rodriguez I, Sweetnam PM, Rubinstein A, Volsky DJ (1994). HIV type 1 infection of human cortical neuronal cells: Enhancement by select neuronal growth factors. AIDS Res Hum Retroviruses.

[35] Nath A (2002). Human immunodeficiency virus (HIV) proteins in neuropathogenesis of HIV dementia. J Infect Dis.

[36] Neumann M, Afonina E, Ceccherini-Silberstein F, Schlicht S, Erfle V, Pavlakis GN (2001). Nucleocytoplasmic transport in human astrocytes: Decreased nuclear uptake of the HIV Rev shuttle protein. J Cell Sci.

[37] Albright AV, Strizki J, Harouse JM, Lavi E, O’connor M, Gonzalez-Scarano F (1996). HIV-1 infection of cultured human adult oligodendrocytes. Virology.

[38] Codazzi F, Menegon A, Zacchetti D, Ciardo A, Grohovaz F, Meldolesi J (1995). HIV-1 gp120 glycoprotein induces [Ca^2+^], responses not only in type-2 but also type-1 astrocytes and oligodendrocytes of the rat cerebellum. Eur Neurosci Assoc.

[39] Anderson E, Zink W, Xiong H, Gendelman HE (2002). HIV-1-associated dementia: A metabolic encephalopathy perpetrated by virus infected and immune-competent mononuclear phagocytes. J Acquir Immune Defic Syndr.

[40] Kaul M, Garden GA, Lipton SA (2001). Pathways to neuronal injury and apoptosis in HIV-associated dementia. Nature.

[41] Albright AV, Shieh JT, O’Connor MJ, Gonzalez-Scarano F (2000). Characterization of cultured microglia that can be infected by HIV-1. J Neurovirol.

[42] Watkins BA, Dorn HH, Kelly WB, Armstrong RC, Potts BJ, Michaels F (1990). Specific tropism of HIV-1 for microglial cells in primary human brain cultures. Science.

[43] Ioannidis JP, Reichlin S, Skolnik PR (1995). Long-term productive human immunodeficiency virus-1 infection in human infant microglia. Am J Pathol.

[44] McCarthy M, He J, Wood C (1998). HIV-1 strain-associated variability in infection of primary neuroglia. J Neurovirol.

[45] Sundar KS, Kamaraju LS, Dingfelder J, McMahon J, Gollapudi S, Wilson WH (1995). β-endorphin enhances the replication of neurotropic human immunodeficiency virus in fetal perivascular microglia. J Neuroimmunol.

[46] Jordan CA, Watkins BA, Kufta C, Dubois-Dalcq M (1991). Infection of brain microglial cells by human immunodeficiency virus type 1 is CD4 dependent. J Virol.

[47] Albright AV, Shieh JT, Itoh T, Lee B, Pleasure D, O’Connor MJ (1999). Microglia express CCR5, CXCR4, and CCR3, but of these, CCR5 is the principal coreceptor for human immunodeficiency virus type 1 dementia isolates. J Virol.

[48] Martin-Garcia J, Kolson DL, Gonzalez-Scarano F (2002). Chemokine receptors in the brain: Their role in HIV infection and pathogenesis. AIDS.

[49] Albright AV, Vos RM, Gonzales-Scarano F (2004). Low-level HIV replication in mixed glial cultures is associated with alterations in processing of p55(Gag). Virology.

[50] Wiley CA, Schrier RD, Nelson JA, Lampert PW, Oldstone MB (1986). Cellular localization of human immunodeficiency virus infection within the brains of acquired immune deficiency syndrome patients. Proc Natl Acad Sci USA.

[51] Takahashi K, Wesselingh SL, Griffin DE, McArthur JC, Johnson RT, Glass JD (1996). Localization of HIV-1 in human brain using polymerase chain reaction/*in situ* hybridization and immunocytochemistry. Ann Neurol.

[52] Fischer-Smith T, Croul S, Adeniyi A, Rybicka K, Morgello S, Khalili K (2004). Macrophage/microglial accumulation and proliferating cell nuclear antigen expression in the central nervous system in human immunodeficiency virus encephalopathy. Am J Pathol.

[53] Dong Y, Benveniste EN (2001). Immune function of astrocytes. Glia.

[54] Brack-Werner R (1999). Astrocytes: HIV cellular reservoirs and important participants in neuropathogenesis. AIDS.

[55] Ranki A, Nyberg M, Ovod V, Haltia M, Elovaara I, Raininko R (1995). Abundant expression of HIV Nef and Rev proteins in brain astrocytes *in vivo* is associated with dementia. AIDS.

[56] Haase AT (1986). Pathogenesis of lentivirus infections. Nature.

[57] Peluso R, Haase A, Stowring L, Edwards M, Ventura P (1985). A Trojan Horse mechanism for the spread of visna virus in monocytes. Virology.

[58] Stins MF, Pearce D, Di Cello F, Erdreich-Epstein A, Pardo CA, Kim KS (2003). Induction of intercellular adhesion molecule-1 on human brain endothelial cells by HIV-1 gp120: Role of CD4 and chemokine coreceptors. Lab Invest.

[59] Petito CK, Cash KS (1992). Blood-brain barrier abnormalities in the acquired immunodeficiency syndrome: Immunohistochemical localization of serum proteins in postmortem brain. Ann Neurol.

[60] Mukhtar M, Harley S, Chen P, BouHamdan M, Patel C, Acheampong E (2002). Primary isolated human brain microvascular endothelial cells express diverse HIV/SIV-associated chemokine coreceptors and DC-SIGN and L-SIGN. Virology.

[61] Kramer-Hammerle S, Rothenaigner I, Wolff H, Bell JE, Brack-Werner R (2005). Cells of the central nervous system as targets and reservoirs of the human immunodeficiency virus. Virus Res.

[62] Bomsel M (1997). Transcytosis of infectious human immunodeficiency virus across a tight human epithelial cell line barrier. Nat Med.

[63] Banks WA, Freed EO, Wolf KM, Robinson SM, Franko M, Kumar VB (2001). Transport of human immunodeficiency virus type 1 pseudoviruses across the blood-brain barrier: Role of envelope proteins and adsorptive endocytosis. J Virol.

[64] Boven LA, van der Bruggen T, Sweder van Asbeck B, Marx JJ, Nottet HS (1999). Potential role of CCR5 polymorphism in the development of AIDS dementia complex. FEMS Immunol Med Microbiol.

[65] Panek RB, Benveniste EN (1995). Class II MHC gene expression in microglia.Regulation by the cytokines IFN-gamma, TNFalpha, and TGF-beta. J Immunol.

[66] Adamson DC, Wildemann B, Sasaki M, Glass JD, McArthur JC, Christov VI (1996). Immunologic NO synthase: Elevation in severe AIDS dementia and induction by HIV-1 gp41. Science.

[67] Minagar A, Shapshak P, Fujimura R, Ownby R, Heyes M, Eisdorfer C (2002). The role of macrophage/microglia and astrocytes in the pathogenesis of three neurologic disorders: HIV-associated dementia, Alzheimer disease, and multiple sclerosis. J Neurol Sci.

[68] Wilt SG, Milward E, Zhou JM, Nagasato K, Patton H, Rusten R (1995). *In vitro* evidence for a dual role of tumor necrosis factor-alpha in human immunodeficiency virus type 1 encephalopathy. Ann Neurol.

[69] Wesselingh SL, Takahashi K, Glass JD, McArthur JC, Griffin JW, Griffin DE (1997). Cellular localization of tumor necrosis factor mRNA in neurological tissue from HIV-infected patients by combined reverse transcriptase/polymerase chain reaction *in situ* hybridization and immunohistochemistry. J Neuroimmunol.

[70] Fiala M, Rhodes RH, Shapshak P, Nagano I, Martinez-Maza O, Diagne A (1996). Regulation of HIV-1 infection in astrocytes: Expression of Nef, TNF-alpha and IL-6 is enhanced in coculture of astrocytes with macrophages. J Neurovirol.

[71] Yoshioka M, Bradley WG, Shapshak P, Nagano I, Stewart RV, Xin KQ (1995). Role of immune activation and cytokine expression in HIV-1-associated neurologic diseases. Adv Neuroimmunol.

[72] Griffin DE (1997). Cytokines in the brain during viral infection: Clues to HIV-associated dementia. J Clin Invest.

[73] Verma AS, Singh A, Singh UP, Dwivedi PD, Gaur RK, Sharma P, Pratap R, Sharma KP, Sharma M (2010). NeuroAIDS in Indian Scenario. Recent trends in biotechnology and microbiology.

[74] Epstein LG, Kuiken C, Blumberg BM, Hartman S, Sharer LR, Clement M (1991). HIV-1 V3 domain variation in brain and spleen of children with AIDS: Tissue-specific evolution within host-determined quasispecies. Virology.

[75] Kodama T, Mori K, Kawahara T, Ringler DJ, Desrosiers RC (1993). Analysis of simian immunodeficiency virus sequence variation in tissues of rhesus macaques with simian AIDS. J Virol.

[76] Korber BT, Kunstman KJ, Patterson BK, Furtado M, McEvilly MM, Levy R (1994). Genetic differences between blood- and brain-derived viral sequences from human immunodeficiency virus type 1-infected patients: Evidence of conserved elements in the V3 region of the envelope protein of brain-derived sequences. J Virol.

[77] Reddy RT, Achim CL, Sirko DA, Tehranchi S, Kraus FG, Wong-Staal F (1996). Sequence analysis of the V3 loop in brain and spleen of patients with HIV encephalitis. AIDS Res Hum Retroviruses.

[78] Wong JK, Ignacio CC, Torriani F, Havlir D, Fitch NJ, Richman DD (1997). *In vivo* compartmentalization of hum an immunodeficiency virus: Evidence from the examination of pol sequences from autopsy tissues. J Virol.

